# Hay-Fever and Its Treatment

**Published:** 1911-09

**Authors:** 


### Hay-Fever and Its Treatment

Beverley Robinson writes in *Merck's Archives* for May, 1911, upon this topic.

Speaking generally, the writer asserts he has had more satisfaction from a combination of camphor, oleoresin of cubeb, glycerin, and petrolatum than from any other local application. The above ingredients, when mixed, are in the form of a relatively soft ointment. They may be used, sniffed well up into the nasal passages, several times a day, and introduced therein either with the end of the finger or on a camel's-hair brush, before being drawn well up and backward into the nasal passages, and when they are felt in the nasopharynx the excess of ointment is hawked down and expectorated from the mouth. At present he is making use of the liquid petrolatum, and the ingredients become a thick, oily liquid instead of a soft ointment. These are sprayed by means of a glass atomiser into the nasal passages several times daily, or whenever relief seems to be much needed. The precise formula now employed by him is:

℞ Pulv. camphoræ, gr. x;

Oleoresinæ cubebæ, m. xx;

Glycerini, f℥j;

Petrolati liq., q. s. ad f℥ss.

M.

In conclusion he adds that in his observation there is no health resort in the East which will invariably, and during successive seasons, accord absolute immunity to attacks of hay-fever. Further, this is also true of a sea voyage, even though it be a long one.—*Therapeutic Gazette.*

